# Finding “Time” for recertification

**DOI:** 10.1051/ject/2025036

**Published:** 2025-09-15

**Authors:** Anthony G. Shackelford

**Affiliations:** 1 Department of Perfusion Services, Medical University of South Carolina Charleston SC USA

Dear Editor,

First let us applaud the American Board of Cardiovascular Perfusion (ABCP) with its current and past directors for a job well done. Through their diligence and hard work in addressing barriers to certification in the ever-changing clinical landscape, the designated Certified Clinical Perfusionist (CCP) has remained a recognized standard in America. Some examples of the adaptations include, in the mid-1990s, we adapted our recertification “case count” criteria due to the impact of Off-Pump Coronary Artery Bypass Grafts (OPCABS). We adjusted and flexed for the Coronavirus (COVID) pandemic and recently added credit for High Fidelity Perfusion Simulation (HFPS). This flexibility and adaptability of the ABCP in meeting these challenges have served the perfusion community and the patients very well. Yet this is a never-ending process as the ABCP continues to monitor the clinical landscape through surveys and other methods, looking for opportunities to assist in meeting its mission. This letter intends is to propose a way to *ADD* to our current recertification process, affording perfusionists to capture more of their clinical practice.

At the time of this writing, the American perfusion community has just passed the annual deadline to submit their clinical and professional activity for recertification. I have seen perfusionists, myself included, almost need until the last day of the annual cycle to get enough cases for recertification. In 2021, the ABCP surveyed CCPs: “Within the last several years, have you struggled to complete your 40-case requirement?”. Forty-seven percent of all CCPs responded to the survey (*n* = 1960). For a variety of reasons, 220 CCPs (11.2%) said “Yes” they did struggle to get their cases (Table 1) [1]. I concur with the variety of responses and submit that this moderate percentage has always been there. For example, in 1991, my pediatric clinical rotation had four CCPs on staff covering approximately 125 cases, but with the high #CCP/case volume ratio every year, they struggled with recertification. Their solution was to visit the large adult hospital in town to obtain sufficient “cases.” In short, any center with high #CCP/case volume ratios or routinely long pump runs requiring multiple perfusionists per case risks struggling for recertification.

**Table 1:** 2021 ABCP survey.

Within the last several years, have you struggled to complete your 40 case requirement? (select all that apply)		
Answer choices	Responses	
No – I don't have any issues completing my 40 case requirement.	88.83%	1741
Yes – Our caseload is down	7.30%	143
Yes – We have more perfusionists than our caseload can cover to remain certified	1.12%	22
Yes – our surgeon(s) mostly perform off-pump cases	1.07%	21
Yes – our TAVR program has reduced our CPB cases	3.11%	61
Yes – but I am able to get cases at other accounts	1.58%	31
Yes – but I have to freelance in order to get cases	0.82%	16
Yes – I don’t work full time	1.17%	23
Other (please elaborate)	2.86%	56
	Answered	1960
	Skipped	266

In 2017, the ABCP wrote, “The uniqueness of the perfusion specialty has the potential to be a constrained resource that cannot be easily replaced” [2]. Well folks, that potential has been realized. Colligan’s paper “Results of the 2019 Survey on the Perceptions of Vacancy and Turnover Among Perfusionists in the United States” revealed a population-wide vacancy rate in perfusion of 12.3% [3]. At this level, it is classified as “severe” (≥11.1%), a level that is most likely above that of registered nurses. Anecdotally, I believe we can all agree that in 2024, we are still experiencing a high vacancy rate. But what does recertification have to do with our shortage of perfusionists? I have seen many competent perfusionists who may need to reduce their work hours to less than full-time, (relocating, military deployments, births in the family, illness, accidents, deaths in the family, etc.) but end up choosing to leave the field early. Why? Not because they have lost their passion for perfusion, but rather because of the “struggle” of not getting enough qualified “cases” for recertification. Interestingly, the work of Colligan showed that having opportunities for “Flex Work” and “Part-time Work” are ways to retain retirees and decrease vacancy and turnover respectively [3].

In this ever-changing environment, I submit that now is one of those times, as in the past, for the ABCP to consider revising or enhancing the ABCP recertification process.

According to the ABCP, to receive clinical case credit the perfusionist must be responsible for the conduct of perfusion for 60% of the “case” [4]. What about the other 40%? Can that percentage of time “count” toward clinical practice when the second perfusionist either started or finished the case? To be blunt, if the 40% perfusionist makes a significant error, they are the one who will be held responsible by the employer and will surely be named in the lawsuit. For the sake of the argument, what if that 40% is longer than a typical 70-minute CABG? I believe here is our opportunity to enhance our current system to help reduce or eliminate those who struggle to get enough cases for recertification while potentially helping with the supply of perfusionists.

So, what is the enhancement? First, to be clear, we should keep the case counting method as a case is a case. Please consider that we have all seen car manufacturers offer warranties with the following conditions: 3 years or 30,000 miles – whichever comes first. I propose we modify their approach to reduce or even eliminate the 11.2% increase. Let us enhance our system this way by letting every minute of bypass count! Perhaps call it “Clinical Minutes” (CM)? Consider we have been doing this under the surface all along with the 60% of minutes of bypass, or the 4-hour with initiation or 6-hour ECMO case credits. This approach would capture all the perfusionist’s “Clinical Perfusion Activity” (CPA) for the CCP’s report, if necessary.

What are the benefits? With a known CM target, there are more opportunities to get CPA, especially in that the longer case with a second perfusionist in the 40% scenario. Otherwise, we are ignoring the 2nd perfusionist’s valuable CPA. With CM, the CCP, the employer, and the rest of the team know the target and can work to achieve that goal together. A win-win and a win for the profession, as this will help retain perfusionists. Another group this will be more helpful for is our Perfusion Educators, to remain clinically certified, as they, too, will use the target number. Lastly, Clinical Minutes could be helpful at a minimum, reducing stress when big life events occur, planned and unplanned. Yes, the ABCP has a process for these, but this would help simplify and reduce the number of perfusionists using that pathway.

The next question: “How many minutes constitute a case credit, or what level of minutes would be enough to qualify for recertification?” I propose we ask the Society of Thoracic Surgeons to query their database and provide us with the following question: “What is the most prevalent adult procedure using cardiopulmonary bypass?” My guess is CABG × 3. Second, ask, “For that type of case, what is the most common *length* of time on cardiopulmonary bypass?” (the mode, not the average). Then simply multiply this value by 40. We then would have a threshold, in quantifiable and readily accessible minutes, that aligns with the ABCP goals. And this number is validated as it represents the typical CPA in America. And lastly circling back to the car warranty analogy, if implemented, the recertification criteria could be 40 (25/15) cases or “X” CM. For completeness, so as not to game the system, we will have put some criteria for a minimum level of CM per case for the minutes to count towards the recertification total. Perhaps half of whatever number the STS gives us (35”). This way someone could not submit a big series of 15-minute CM.

Reiterating, I am not suggesting we abandon case counting, nor is this suggestion or parts thereof the only approach. Additionally, I do not suggest using this for any other procedures (HYPEC, NRP, etc.). However, 11% of any community is a significant number. And it appears it will remain, if not increase. If we wish to lower or eliminate this percentage, then more pathways to recertification need to be added. This method is not drastically different from what we are already doing. It maintains the CCP's representation of what it has always been while addressing these barriers. This method will help the ABCP meet its mission regarding the CCP certification while reducing the “the struggle” for the community it serves. I am asking perfusionists to join me in requesting that the ABCP consider this method. And do what it has always done when barriers arise. Lastly, I would be very honored to assist the ACBP in navigating this project.

Thank you for your “time!”
